# Assessing the Significance of Interleukin-6, Interleukin-10, and Tumor Necrosis Factor-Alpha in Peri-Implantitis Development: A Biomarker Study

**DOI:** 10.7759/cureus.90192

**Published:** 2025-08-15

**Authors:** Shreya Bhukal, Rupali Malik, Rukshana Begum, Kavya Adapala, Manikesh Killamsetty, Palak Arora, Seema Gupta

**Affiliations:** 1 Department of Public Health Dentistry, All India Institute of Medical Sciences, New Delhi, IND; 2 Department of Public Health Dentistry, Teerthanker Mahaveer Dental College and Research Centre, Moradabad, IND; 3 Department of Oral Medicine and Radiology, Kalinga Institute of Dental Sciences, Bhubaneswar, IND; 4 Department of Periodontics, Bharati Vidyapeeth (Deemed to be University) Dental College and Hospital, Sangli, IND; 5 Department of Oral and Maxillofacial Surgery, Kalinga Hospitals, Bhubaneswar, IND; 6 Department of Prosthodontics, Institute of Dental Studies and Technologies, Modinagar, IND; 7 Department of Orthodontics, Kothiwal Dental College and Research Centre, Moradabad, IND

**Keywords:** biomarkers, cytokines, dental implants, inflammation, peri-implantitis

## Abstract

Introduction: Peri-implantitis is an inflammatory condition involving soft tissue and bone loss around dental implants. Traditional diagnostic methods may miss early signs, leading to interest in using molecular markers for earlier and more accurate detection. This study investigated the roles of interleukin-6 (IL-6), interleukin-10 (IL-10), and tumor necrosis factor-alpha (TNF-α) in peri-implantitis by comparing their levels in peri-implant crevicular fluid (PICF) between healthy implants and peri-implantitis groups. Therefore, this study aimed to clarify the diagnostic potential of this method in periodontally healthy patients.

Materials and methods: A prospective, cross-sectional study was conducted on 64 systemically healthy adults (aged 18-70 years), equally divided into Group 1 with healthy implant (probing depth <4 mm, no bleeding on probing, no marginal bone loss) and Group 2 with peri-implantitis (probing depth ≥4 mm, bleeding on probing, marginal bone loss ≥2 mm) groups. The exclusion criteria were smoking (>10 pack-years), recent antibiotic use, pregnancy, and immunosuppressive conditions. The groups were matched for age, sex, and implant characteristics (Straumann, Basel, Switzerland). Clinical assessments were performed using an UNC-15 periodontal probe (Hu-Friedy; Chicago, IL, USA). PICF was collected using periopaper strips (Oraflow Inc., Smithtown, NY, USA) and stored in Corning tubes (Corning Inc., Corning, NY, USA). Cytokine levels were quantified using human IL-6, IL-10, and TNF-α enzyme-linked immunosorbent assay (ELISA) kits (R&D Systems, Minneapolis, MN, USA) on a SpectraMax M5 reader (Molecular Devices, San Jose, CA, USA). The data were subjected to statistical analyses.

Results: The peri-implantitis group showed significantly higher plaque index (2.66 ± 0.40) and probing depth (4.85 ± 0.41 mm) compared to the healthy implant group (plaque index: 0.96 ± 0.27; probing depth: 3.09 ± 0.38 mm). Cytokine levels in PICF were markedly elevated in peri-implantitis cases: IL-6 (235.56 ± 56.34 pg/mL vs. 22.88 ± 8.06 pg/mL), IL-10 (73.13 ± 15.11 pg/mL vs. 13.13 ± 4.25 pg/mL), and TNF-α (148.94 ± 53.99 pg/mL vs. 8.31 ± 2.89 pg/mL). Logistic regression identified plaque index (OR = 162.37), probing depth (OR = 33.57), and smoking (OR = 20.50) as strong predictors of peri-implantitis, while longer loading time, male sex, and posterior implant location showed protective associations.

Conclusion: IL-6, IL-10, and TNF-α levels were significantly elevated in patients with peri-implantitis, supporting their diagnostic potential. Plaque control and smoking cessation are critical for prevention, and biomechanical factors influence the risk.

## Introduction

Peri-implantitis, a chronic inflammatory condition affecting dental implants, is characterized by progressive bone loss and soft tissue inflammation, which pose significant challenges to the long-term success of implants [1]. The worldwide incidence of implant failure ranges from 1.9% to 3.6% [2]. Implant failures can be classified into two categories based on their occurrence: early and late failures [3]. Peri-implantitis is one of the main reasons for implant failure; therefore, reliable diagnostic and prognostic tools are required to guide clinical management [1]. Current diagnostic methods rely heavily on clinical and radiographic parameters, such as probing depth and marginal bone loss, but lack specificity for early detection and progression monitoring [4]. Inflammatory biomarkers, particularly cytokines, offer a promising avenue for understanding the immunological mechanisms of peri-implantitis and improving diagnostic precision [5]. This study focuses on three cytokines, interleukin-6 (IL-6), interleukin-10 (IL-10), and tumor necrosis factor-alpha (TNF-α), owing to their controversial roles in peri-implantitis, with the aim of clarifying their diagnostic and prognostic utility in distinguishing healthy implants from diseased states [5,6].

The need for this study stems from the limitations of existing diagnostic approaches and inconsistent findings regarding cytokine biomarkers in peri-implantitis [5,6]. While proinflammatory cytokines, such as interleukin-1beta (IL-1β), are consistently elevated in the peri-implant crevicular fluid (PICF) and saliva of affected patients [7], the roles of IL-6, IL-10, and TNF-α are less clear, with studies reporting conflicting results [5,6,8]. For instance, IL-6, a key mediator of inflammation and bone resorption, was elevated in PICF and saliva in a previous study [9]; however, another study found no significant differences [10]. Similarly, TNF-α, which is critical for bone remodeling, shows elevated levels in peri-implantitis [5] but lacks a consistent correlation with clinical parameters, such as probing depth, raising questions about its specificity [6]. IL-10 is a cytokine found in humans that modulates both immunoregulatory and inflammatory processes in the body. It possesses notable anti-inflammatory characteristics, which are essential for attenuating the immune response to infectious agents, thus mitigating potential harm to the host [11]. Ghighi et al. [12] conducted a study that demonstrated a substantial elevation in IL-10 levels within tissues affected by peri-implantitis. In contrast, Severino et al. [13] found no statistically significant alterations in the levels of IL-10 across different experimental groups. These inconsistencies highlight the need to investigate biomarkers to resolve their roles and enhance the diagnostic strategies.

The selection of IL-6, IL-10, and TNF-α in this study was driven by their controversial roles in peri-implantitis. Unlike IL-1β and IL-8, which show consistent elevation [8], these cytokines exhibit variable expression across studies and are influenced by factors such as disease stage, sample type (PICF vs. saliva), and patient-specific variables (such as systemic conditions). For example, a review noted that IL-6’s diagnostic reliability is debated owing to inconsistent correlations with bone loss, while the local versus systemic significance of TNF-α remains unclear [14]. The variable expression of IL-10 is reduced in some cases but unchanged or elevated in others, further underscoring its controversial prognostic value [12,13]. By focusing on these biomarkers, this study aimed to clarify their expression patterns in healthy and peri-implantitis patients using enzyme-linked immunosorbent assay (ELISA) to quantify their levels in PICF. Ultimately, this study aimed to fill critical gaps in peri-implantitis research, offer insights into immune dysregulation, and support the development of targeted diagnostic tools.

## Materials and methods

This prospective, cross-sectional study was conducted at the Department of Public Health Dentistry, Teerthanker Mahaveer Dental College and Research Centre, Moradabad, India, between April 2022 and April 2024. The study protocol was approved by the Institutional Ethical Committee (TMDCRC/IEC/21-22/PPD21) and adhered to the principles of the Declaration of Helsinki (World Medical Association, 2013). Written informed consent was obtained from all participants prior to enrolment, detailing the study’s purpose, procedures, risks, benefits, and their right to withdraw at any time without any consequences. Consent forms were provided in English and explained by the trained study personnel.

The sample size for this study was calculated to achieve 95% statistical power with a 5% alpha error threshold, based on an effect size of 0.82 for TNF-α differences between healthy and peri-implantitis tissue [15]. Power analysis determined that a minimum of 32 participants per group was required to ensure robust intergroup comparisons. All calculations were performed using G*Power 3.1.9.2 (Heinrich Heine University, Düsseldorf, Germany).

Eligible participants (n = 64) were systemically healthy adults aged 18-70 years. The healthy implant group consisted of individuals with at least one functional single unit dental implant, no clinical or radiographic signs of peri-implantitis or mucositis (probing depth < 4 mm, no bleeding on probing, and no marginal bone loss beyond physiological remodeling), and no history of periodontal disease. The peri-implantitis group included individuals with at least one single unit dental implant diagnosed with peri-implantitis, defined as a probing depth ≥ 4 mm, presence of bleeding on probing, and radiographic marginal bone loss ≥ 2 mm beyond the first year post-implantation, according to the 2017 European Federation of Periodontology and American Academy of Periodontology (EFP/AAP) criteria [16]. Both groups must be periodontally healthy (no gingivitis or periodontitis in natural teeth, defined as probing depth ≤ 3 mm, no bleeding on probing, and no clinical attachment loss). The exclusion criteria included the use of antibiotics or anti-inflammatory drugs within the last three months, current smokers or those with >10 pack-years, pregnant or lactating women, individuals undergoing immunosuppressive therapy or with conditions affecting cytokine levels (such as rheumatoid arthritis), and patients who had undergone peri-implant treatment in the area to be evaluated.

To manage confounders, implant characteristics (such as titanium and moderately rough surfaces; Straumann, Basel, Switzerland) were standardized and documented in the patient records. Periodontal history was verified through full-mouth examinations and patient records to confirm the absence of periodontitis or successful treatment (no active disease for two or more years). Systemic health was assessed through medical records and complete blood tests. Oral hygiene was standardized with professional cleaning one week prior to the study and verified using the Plaque Index as PI (score ≤ 2) [17].

Clinical assessments included probing depth and bleeding on probing, measured using a UNC-15 periodontal probe (Hu-Friedy, Chicago, IL, USA). Periapical radiographs were analyzed using ImageJ software (National Institutes of Health, Bethesda, MD, USA) for any crestal bone loss. PICF was collected using periopaper strips (Oraflow Inc., Smithtown, NY, USA) inserted into the peri-implant sulcus for 30 s in sterile tubes (Corning Inc., Corning, NY, USA). The samples were stored at -80^0^C until analysis.

**Figure 1:**
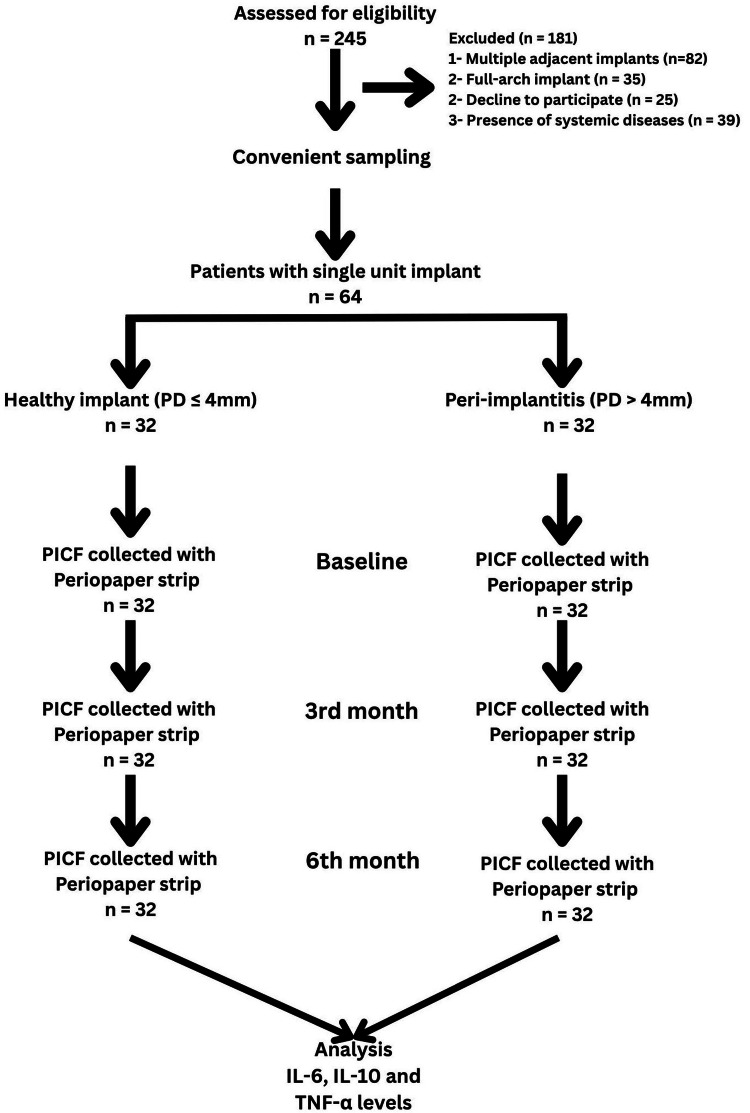
Study flow chart. PD: Probing depth, PICF: Peri-implant crevicular fluid, IL-6: Interleukin-6, IL-10: Interleukin-10, TNF-α: Tumor necrosis factor-alpha.

Cytokine levels were quantified using human IL-6, IL-10, and TNF-α ELISA kits (R&D Systems, Minneapolis, MN, USA). Assays were performed according to the manufacturer’s protocols, with samples diluted in assay buffer, and absorbance was measured at 450 nm using a SpectraMax M5 microplate reader (Molecular Devices, San Jose, CA, USA). The microplate reader was calibrated using a standard calibration plate (Molecular Devices) before each run to ensure an accuracy within ±2%. Inter-assay and intra-assay coefficients of variation were maintained below 10% with duplicate measurements to ensure reliability. The cytokines' levels were checked at multiple time intervals (third month and sixth month) as given in the study flow chart (Figure 1). Clinical examiners were calibrated to achieve an intra-class correlation coefficient of ≥ 0.85 for probing depth and bleeding on probing measurements, which were assessed during a pilot phase with 10 non-study participants.

Statistical analyses were performed using SPSS Statistics version 26 (IBM Corp., Armonk, NY, USA). Categorical variables (sex, smoking, alcohol, and implant location) were presented as frequencies and percentages and compared between the healthy implant and peri-implantitis groups using the chi-square test of independence. Continuous variables (age, post-implant loading duration, loading time, PI, probing depth, IL-6, IL-10, and TNF-α) were assessed for normality using the Shapiro-Wilk test. For normally distributed variables, intergroup comparisons were performed using independent t-tests, with effect sizes reported as Cohen’s d values. Logistic regression analysis was conducted to identify predictors of peri-implantitis outcomes, adjusting for confounders (age, sex, smoking, PI, implant location, post-implant loading duration, and loading time), with odds ratios, standard errors, and p-values. A two-tailed p-value of < 0.05 was considered statistically significant for all analyses.

## Results

Chi-square analysis revealed no statistically significant associations between any of the demographic variables and peri-implantitis status. While the peri-implantitis group showed a higher proportion of males, this difference was not statistically significant (χ² = 2.33, p = 0.127). Similarly, the smoking prevalence was numerically higher in the peri-implantitis group, but this difference was not significant (χ² = 1.64, p = 0.2). Alcohol consumption patterns showed minimal variation between the groups. Implant location distribution was comparable between the groups, with posterior implants being more common in both healthy and peri-implantitis cases (χ² = 0.27, p = 0.599). These findings suggest that, within our study population, neither sex, smoking habits, alcohol consumption, nor implant location demonstrated a statistically significant association with peri-implantitis development (Table 1).

**Table 1:** Analysis of independent association for baseline characteristics between healthy implant and peri-implantitis groups with Chi square test. p > 0.05 denotes no statistical significance using chi-square test of independence. Data are presented as frequency (n) and percentage (%), where n denotes number of participants in each group.

Variables	Category	Healthy (n = 32, 100%)		Peri-implantitis (n = 32, 100%)		Chi square (χ²) value	p-value
		n	%	n	%		
Sex	Female	16	50.0	10	31.2	2.33	0.127
	Male	16	50.0	22	68.7		
Smoking	No	28	87.5	24	75.0	1.64	0.201
	Yes	4	12.5	8	25.0		
Alcohol	No	24	75.0	22	68.7	0.30	0.578
	Yes	8	25.0	10	31.3		
Location of implant	Anterior	10	31.3	12	37.5	0.27	0.599
	Posterior	22	68.7	20	62.5		

Independent t-test analysis revealed significant differences between the healthy and peri-implantitis groups for several key variables. Although age, implant duration, and loading time showed no significant differences, all clinical and biological markers demonstrated highly significant variations (p < 0.001). The peri-implantitis group exhibited substantially higher PI scores (2.66 ± 0.40 vs. 0.96 ± 0.27 respectively in peri-implantitis and healthy group, d = 4.9) and probing depths (4.85 ± 0.41 mm vs. 3.09 ± 0.38 mm respectively in peri-implantitis and healthy group, d = 4.4), confirming the clinical diagnosis criteria. Most notably, cytokine levels were dramatically elevated in peri-implantitis cases: IL-6 showed a 10-fold increase (235.56 ± 56.34 pg/mL vs. 22.88 ± 8.06 pg/mL respectively in peri-implantitis and healthy group, d = 5.2), IL-10 levels were 5.5 times higher (73.13 ± 15.11 pg/mL vs. 13.13 ± 4.25 pg/mL respectively in peri-implantitis and healthy group, d = 5.4), and TNF-α demonstrated an 18-fold elevation (148.94 ± 53.99 pg/mL vs. 8.31 ± 2.89 pg/mL respectively in peri-implantitis and healthy group, d = 3.6). The large effect sizes underscored the substantial biological differences between groups. These findings strongly support the role of inflammatory cytokines in the pathogenesis of peri-implantitis and validate their potential as diagnostic biomarkers (Table 2).

**Table 2:** Intergroup comparison of clinical and biomarker variables between healthy implant and peri-implantitis groups using independent t-test. *p < 0.05 denotes statistical significance using independent t-test. Data are presented as mean, standard deviation, and 95% confidence interval of the mean.

Variables	Groups	Mean	Standard Deviation	95% Confidence Interval (Mean)		t value	p-value	Effect size (Cohen’s d)
				Lower limit	Upper limit			
Age (years)	Healthy	40.18	6.37	37.88	42.48	-0.9	0.37	0.22
	Peri-implantitis	41.62	6.36	39.33	43.91			
Post-loading duration (months)	Healthy	9.81	1.26	9.35	10.27	1.1	0.274	0.27
	Peri-implantitis	9.46	1.22	9.02	9.91			
Loading time (months)	Healthy	3.18	0.64	2.95	3.42	-1.19	0.236	0.29
	Peri-implantitis	3.37	0.60	3.15	3.59			
Plaque Index	Healthy	0.96	0.27	0.86	1.06	-19.8	0.001*	4.9
	Peri-implantitis	2.65	0.40	2.51	2.80			
Probing depth (mm)	Healthy	3.09	0.38	2.95	3.23	-17.8	0.001*	4.4
	Peri-implantitis	4.85	0.40	4.70	4.99			
Interleukin-6 (pg/mL)	Healthy	22.87	8.05	19.97	25.77	-21.1	0.001*	5.2
	Peri-implantitis	235.56	56.33	215.25	255.87			
Interleukin-10 (pg/mL)	Healthy	13.12	4.24	11.59	14.65	-21.6	0.001*	5.4
	Peri-implantitis	73.12	15.11	67.67	78.57			
Tumor necrosis factor-alpha (pg/mL)	Healthy	8.31	2.89	7.27	9.35	-14.7	0.00*	3.6
	Peri-implantitis	148.93	53.98	129.47	168.40			

Logistic regression analysis revealed several significant predictors of peri-implantitis. Smoking (OR = 20.502) and PI (OR = 16.372) were the strongest risk factors, followed by probing depth (OR = 13.568). Conversely, longer loading time showed a protective effect (OR = 0.119). Among the cytokines, IL-10 demonstrated a positive association (OR = 1.466), whereas TNF-α showed a marginal negative association (OR = 0.988). Posterior implant location (odds ratio [OR] = 0.587) and male sex (OR = 0.356) were protective factors. The dramatic effect size of the PI highlights its crucial role in disease pathogenesis, while the strong association with smoking reinforces its modifiable risk factor status. The protective effects of posterior implants may reflect biomechanical advantages or sampling biases. The cytokine patterns suggest a potential role for IL-10 as a disease marker, although its elevation may represent a compensatory anti-inflammatory response. These findings emphasize the multifactorial nature of peri-implantitis, where traditional clinical parameters (plaque and probing depth) outweigh the predictive value of the molecular markers (Table 3).

**Table 3:** Logistic regression analysis for predictors of peri-implantitis outcome. Estimates represent unstandardized coefficients, with odds ratios indicating the change in odds per unit increase in the predictor, Model adjusted for all listed variables.

Variables	Estimate	Standardized Coefficient	Odds Ratio (OR)
Interleukin-6 (pg/mL)	0.105	11.995	1.111
Interleukin-10 (pg/mL)	0.382	12.302	1.466
Tumor necrosis factor-alpha (pg/mL)	-0.012	-0.956	0.988
Implant location (Posterior)	-0.532	-0.084	0.587
Smoking (Yes)	3.021	-1.337	20.502
Sex (Male)	-1.033	4.676	0.356
Post-loading duration (months)	-0.068	3.399	0.935
Loading time (months)	-2.125	-0.532	0.119
Plaque Index (score)	5.09	3.021	16.372
Probing depth (mm)	3.514	-1.033	13.568

## Discussion

The results of this study provide significant insights into the roles of IL-6, IL-10, and TNF-α in peri-implantitis, addressing their controversial status as biomarkers. The findings revealed no significant demographic differences (sex, smoking, alcohol, and implant location) between the groups, significant elevations in clinical parameters (PI and probing depth), cytokines in the peri-implantitis group, and strong predictive roles for smoking, PI, and probing depth in logistic regression, with nuanced cytokine contributions.

Chi-square analysis showed no significant association between demographic variables and peri-implantitis status. This finding suggests that these factors did not significantly influence the development of peri-implantitis in our cohort. The lack of an association with smoking, despite its higher prevalence in the peri-implantitis group, contrasts with a previous study by Martinez-Amargant et al. [18], who reported a significant association between smoking and peri-implant disease. This discrepancy may stem from our strict exclusion criteria (smokers with >10 pack-years excluded), which reduced the smoking exposure of the sample and thus its impact. One study reported that the incidence of peri-implantitis was higher in patients who smoked for more than 21 years [18]. Similarly, the absence of a sex difference aligns with a previous study that reported no sex-based predisposition to crestal bone loss and peri-implantitis [19]. The groups were matched for sex, potentially masking these effects on the results.

Independent t-test analysis demonstrated notable differences in the clinical and biomarker variables between the healthy implant and peri-implantitis groups. The PI, probing depth, and IL-6, IL-10, and TNF-α levels were significantly higher in the peri-implantitis group, with large effect sizes indicating substantial biological differences between the groups. The elevated PI and probing depth were consistent with a previous study that reported a cause-and-effect relationship between dental plaque and peri-implant mucositis [20]. Contemporary research indicates that bacterial colonization occurs within 30 min of implantation [21], achieving stability after an interval of two weeks [22].

The dramatic cytokine elevations found in our study are consistent with those reported in previous studies [7-9]. Duarte et al. [23] found that IL-6 and TNF-α levels were significantly higher in the PICF of patients with peri-implantitis, and Ghighi et al. [12] noted variable expression of IL-10. However, our IL-6 levels (10-fold increase) were higher than those reported by Liskmann et al. [9], who observed moderate elevations, possibly due to our focus on PICF (more localized) versus saliva in their study [9] or our exclusion of systemic confounders such as diabetes, which elevates the baseline cytokines [24]. This 5.5-fold increase in IL-10 levels contrasts with the findings of a previous study by Severino et al. [13], who reported no significant difference in IL-10 levels between the peri-implantitis and healthy implant groups. Our finding of elevated IL-10 levels may reflect a compensatory mechanism in periodontally healthy patients, as our strict inclusion criteria eliminated periodontitis-related inflammation, potentially amplifying the role of IL-10 in counteracting proinflammatory cytokines [11].

The significant elevations in IL-6, IL-10, and TNF-α levels in the peri-implantitis group compared to those in the healthy implant group, as observed in this study, can be attributed to the heightened inflammatory response characteristic of peri-implantitis. Peri-implantitis is characterized by chronic inflammation around dental implants driven by bacterial plaque accumulation, which triggers an immune cascade involving proinflammatory cytokines, such as IL-6 and TNF-α, to combat infection and tissue damage [25]. IL-6, a key mediator of inflammation, is upregulated in response to bacterial challenge, promoting bone resorption and tissue destruction, as reported by Duarte et al. [23]. Similarly, TNF-α, which is critical for osteoclast activation, is elevated in PICF owing to localized inflammation, consistent with the findings of He et al. [7]. Increased IL-10 levels, an anti-inflammatory cytokine, likely reflect a compensatory mechanism to modulate the intense proinflammatory response, as noted in some studies where IL-10 increases to counteract tissue damage in periodontally healthy patients [11,12]. The periodontally healthy status of the peri-implantitis group in this study minimized confounding periodontal inflammation, ensuring that these cytokine elevations were specific to peri-implantitis, driven by local microbial and immune interactions.

Logistic regression analysis revealed that smoking, PI, and probing depth were significant predictors of peri-implantitis, while IL-10 and TNF-α exhibited a positive and slight negative association, respectively. Posterior implant location, male sex, and extended loading time were identified as protective factors against peri-implantitis. The strong effect of the PI aligns with previous studies [20-22], emphasizing plaque as the primary driver of peri-implant inflammation. The high odds for smoking, despite non-significant chi-square results, suggests that its predictive power emerges when combined with other variables, which is consistent with the findings of Martinez-Amargant et al. [18]. The predictive role of probing depth is supported by Benedek et al. [26], who found increased probing depth in patients with peri-implantitis, suggesting a correlation between deeper pockets and disease severity. The positive association with IL-10 is intriguing, in contrast to the findings of Severino et al. [13]. The marginal negative association of TNF-α is unexpected and contrasts with the findings of Jin et al. [5], who found TNF-α to be predictive of peri-implantitis. This may result from multicollinearity with the plaque index or probing depth, which dominates the model or reflects the variable systemic versus local role of TNF-α [24]. The protective effect of posterior implants may be related to biomechanical stability, whereas the protective role of the male sex could reflect hormonal or hygiene differences, although this requires further exploration. The protective effect of a longer loading time suggests adaptation of the peri-implant tissues, aligning with the limited studies on implant duration.

The clinical implications include the potential use of IL-6, IL-10, and TNF-α as diagnostic biomarkers, given their significant elevation, particularly in conjunction with clinical parameters such as PI and probing depth. The elevation of IL-10 levels suggests a compensatory role that could guide targeted therapies (such as anti-inflammatory agents). The strong predictive power of the plaque index and smoking emphasizes the importance of oral hygiene and smoking cessation in preventing peri-implantitis.

However, the limitations of this study include the small sample size, which may limit the statistical power, and the cross-sectional design, which precludes causal inferences. The exclusion of systemic conditions and heavy smokers reduces the generalizability of the results to broader populations. Potential assay variability and a focus on PICF may overlook systemic cytokine patterns. Future longitudinal studies with larger and more diverse cohorts and multi-sample analyses are needed to further validate these findings.

## Conclusions

This prospective cross-sectional study demonstrated that IL-6, IL-10, and TNF-α levels in PICF were significantly elevated in periodontally healthy patients with peri-implantitis compared to those with healthy-periodontal implants. The PI, probing depth, and smoking have emerged as strong predictors of peri-implantitis, underscoring the critical role of oral hygiene and modifiable risk factors. Posterior implant location, male sex, and longer loading time were protective factors, suggesting biomechanical and biological influences on susceptibility to disease. These results support the potential of IL-6, IL-10, and TNF-α as diagnostic biomarkers and highlight the need for targeted interventions focusing on plaque control and smoking cessation to mitigate the risk of peri-implantitis.

## References

[1] Dereka X, Mardas N, Chin S, Petrie A, Donos N (2012). A systematic review on the association between genetic predisposition and dental implant biological complications. Clin Oral Implants Res.

[2] Alsaadi G, Quirynen M, Michiles K, Teughels W, Komárek A, van Steenberghe D (2008). Impact of local and systemic factors on the incidence of failures up to abutment connection with modified surface oral implants. J Clin Periodontol.

[3] Manor Y, Oubaid S, Mardinger O, Chaushu G, Nissan J (2009). Characteristics of early versus late implant failure: a retrospective study. J Oral Maxillofac Surg.

[4] Monje A, Salvi GE (2024). Diagnostic methods/parameters to monitor peri-implant conditions. Periodontol 2000.

[5] Jin Q, Teng F, Cheng Z (2021). Association between common polymorphisms in IL-1 and TNFα and risk of peri-implant disease: a meta-analysis. PLoS One.

[6] Jamshidy L, Tadakamadla SK, Choubsaz P, Sadeghi M, Tadakamadla J (2021). Association of IL-10 and TNF-α polymorphisms with dental peri-implant disease risk: a meta-analysis, meta-regression, and trial sequential analysis. Int J Environ Res Public Health.

[7] He K, Jian F, He T, Tang H, Huang B, Wei N (2020). Analysis of the association of TNF-α, IL-1A, and IL-1B polymorphisms with peri-implantitis in a Chinese non-smoking population. Clin Oral Investig.

[8] La Monaca G, Pranno N, Patini R, Polimeni A, Cordaro M, Cristalli MP (2025). Biomarkers in peri-implant crevicular fluid of healthy implants and those with peri-implant diseases: a systematic review and meta-analysis. J Oral Pathol Med.

[9] Liskmann S, Vihalemm T, Salum O, Zilmer K, Fischer K, Zilmer M (2006). Correlations between clinical parameters and interleukin-6 and interleukin-10 levels in saliva from totally edentulous patients with peri-implant disease. Int J Oral Maxillofac Implants.

[10] Mengel R, Stelzel M, Hasse C, Flores-de-Jacoby L (1996). Osseointegrated implants in patients treated for generalized severe adult periodontitis. An interim report. J Periodontol.

[11] Iyer SS, Cheng G (2012). Role of interleukin 10 transcriptional regulation in inflammation and autoimmune disease. Crit Rev Immunol.

[12] Ghighi M, Llorens A, Baroukh B, Chaussain C, Bouchard P, Gosset M (2018). Differences between inflammatory and catabolic mediators of peri-implantitis and periodontitis lesions following initial mechanical therapy: an exploratory study. J Periodontal Res.

[13] Severino VO, Beghini M, de Araújo MF, de Melo ML, Miguel CB, Rodrigues WF, de Lima Pereira SA (2016). Expression of IL-6, IL-10, IL-17 and IL-33 in the peri-implant crevicular fluid of patients with peri-implant mucositis and peri-implantitis. Arch Oral Biol.

[14] Wang T, He C (2020). TNF-α and IL-6: the link between immune and bone system. Curr Drug Targets.

[15] Zani SR, Moss K, Shibli JA (2016). Peri-implant crevicular fluid biomarkers as discriminants of peri-implant health and disease. J Clin Periodontol.

[16] Berglundh T, Armitage G, Araujo MG (2018). Peri-implant diseases and conditions: consensus report of workgroup 4 of the 2017 World Workshop on the Classification of Periodontal and Peri-Implant Diseases and Conditions. J Clin Periodontol.

[17] Löe H (1967). The Gingival Index, the Plaque Index and the Retention Index systems. J Periodontol.

[18] Martinez-Amargant J, de Tapia B, Pascual A, Takamoli J, Esquinas C, Nart J, Valles C (2023). Association between smoking and peri-implant diseases: a retrospective study. Clin Oral Implants Res.

[19] Banu RF, Kumar VA (2023). Early implant bone loss in the preprosthetic phase: a retrospective study. J Oral Implantol.

[20] Pontoriero R, Tonelli MP, Carnevale G, Mombelli A, Nyman SR, Lang NP (1994). Experimentally induced peri-implant mucositis. A clinical study in humans. Clin Oral Implants Res.

[21] Fürst MM, Salvi GE, Lang NP, Persson GR (2007). Bacterial colonization immediately after installation on oral titanium implants. Clin Oral Implants Res.

[22] Quirynen M, Vogels R, Peeters W, van Steenberghe D, Naert I, Haffajee A (2006). Dynamics of initial subgingival colonization of 'pristine' peri-implant pockets. Clin Oral Implants Res.

[23] Duarte PM, Serrão CR, Miranda TS (2016). Could cytokine levels in the peri-implant crevicular fluid be used to distinguish between healthy implants and implants with peri-implantitis? A systematic review. J Periodontal Res.

[24] Assery NM, Jurado CA, Assery MK, Afrashtehfar KI (2023). Peri-implantitis and systemic inflammation: a critical update. Saudi Dent J.

[25] Fragkioudakis I, Tseleki G, Doufexi AE, Sakellari D (2021). Current concepts on the pathogenesis of peri-implantitis: a narrative review. Eur J Dent.

[26] Benedek C, Kerekes-Máthé B, Bereșescu L (2024). Influencing factors regarding the severity of peri-implantitis and peri-implant mucositis. Diagnostics (Basel).

